# Caerulomycin A inhibits Th2 cell activity: a possible role in the management of asthma

**DOI:** 10.1038/srep15396

**Published:** 2015-10-20

**Authors:** Weshely Kujur, Rama Krishna Gurram, Nazia Haleem, Sudeep K. Maurya, Javed N. Agrewala

**Affiliations:** 1 Immunology Laboratory, CSIR-Institute of Microbial Technology, Chandigarh-160036, INDIA

## Abstract

We have recently demonstrated that Caerulomycin A induces regulatory T cells differentiation by suppressing Th1 cells activity. The role of regulatory T cells is well established in suppressing the function of Th2 cells. Th2 cells are known to inflict the induction of the activation of asthma. Consequently, in the present study, we monitored the influence of Caerulomycin A in inhibiting the activity of Th2 cells and its impact in recuperating asthma symptoms. Interestingly, we observed that Caerulomycin A significantly suppressed the differentiation of Th2 cells, as evidenced by downregulation in the GATA-3 expression. Further, decline in the levels of IL-4, IL-5 and IL-13 cytokines and IgE was noted in the animals suffering from asthma. Furthermore, we noticed substantial suppression in the inflammatory response and number of eosinophils in the lungs. In essence, this study signifies an important therapeutic role of Caerulomycin A in asthma.

Asthma is a chronic pulmonary disease caused by inflammation of the airway mucosa and characterized by breathlessness and wheezing. Approximately 300 million people worldwide are suffering from asthma^1^. Recently, it has been reported that asthma incidents has considerably increased in the Western world^2^. It is estimated that 7% of US citizens suffer from asthma^3^. Thus, causing an increasing financial burden on health care services^4^.

The main cause of asthma is the dysregulated immune response towards harmless environmental antigens. Airway remodelling, the reason behind the disease pathology of asthma is characterized by chronic inflammation of the airway, excessive mucus secretion and subepithelial fibrosis^5^. The airway mucosal system is a constitutive site exposed to microbes and non-microbial foreign substances. The immune system in the airway mucosa efficiently defends against pathogens. The homeostasis in mucosal surface is maintained by a delicate balance between pro- and anti-inflammatory conditions^6^. The disturbance in this balance results in airway hyper-responsiveness, which leads to allergy and asthma generation.

Although, underlying etiology behind the asthma pathogenesis is complex but Th2 cell is considered as a key player in the initiation, progression and persistence of asthma^7^. Th2 cell is a subset of CD4 T cells that mainly secretes IL-4 and IL-13 cytokines^8^. Despite the availability of numerous drugs, corticosteroids are most widely used for the treatment of asthma. Despondently, some individuals do not respond to the existing therapies^9^,^10^. Th2 response is known to induce glucocorticoid resistance^11^. Hence, patients suffering from severe asthma become unresponsive to the corticosteroids. Rise in asthma cases inflict huge economic burden on the healthcare related costs^12^. Consequently, there is an urgent need to search new therapeutic interventions that can inhibit Th2 cell response and eventually cures asthma.

Regulatory T cells (Tregs) are a subset of T helper cells. Tregs play a key role in the maintenance of immune homeostasis by controlling T cell-mediated immune response. There are increasing evidences that Tregs can actively suppress the activity of potentially harmful T cells^13^,^14^. Further, they have been implicated in antagonizing Th2 response and amelioration of allergic diseases^15^,^16^. Recently, we reported the induction of Tregs by Caerulomycin A (CaeA)^17^. Interestingly, the enhancement of Tregs adequately regress the immunopathology caused by Th2 cells. Hence, for this reason wanted to evaluate the influence of CaeA in alleviating asthma. Interestingly, we observed that CaeA significantly suppresses Th2 response and significantly attenuated asthma symptoms.

## Materials and Methods

### Chemicals and reagents

All cytokines and Abs used in ELISA and flowcytometry were procured from BD Biosciences (Franklin Lakes, NJ). FCS and RPMI-1640 were procured from Invitrogen (Carlsbad, CA). L-glutamine, L-pyruvate, penicillin, concanavalin A and streptomycin were from Serva (Heidelberg, Germany). Media components were purchased from Hi-media (Mumbai, India). Caerulomycin A was either procured from LKT Laboratories (St. Paul, MN) or was from actinomycetes, as described elsewhere^18^.

### Mice

Female BALB/c and C3H/HeJ mice (6–8 wk) were procured from the experimental animal facility of the Institute of Microbial Technology. All experiments were reviewed and permitted by the Institutional Animal Ethical Committee and were carried out in accordance with the approved guidelines.

### Differentiation of Th2 cells

Naïve CD4 T cells were purified by magnetic activated cell sorting (MACS). The isolated naïve T cells were highly purified and devoid of non-CD4 T cell population as ascertained by flowcytometry. Purity of naïve CD4 T cells obtained was 96% and devoid of CD8^+^ (CD8 T cells), B220^+^ (B cells), F4/80^+^ (macrophages) and CD11c^+^ (dendritic cells) cells population as ascertained by surface staining with their respective fluorochrome-tagged Abs and monitored through flowcytometry. Naïve CD4 T cells (2 × 10^5^) were stimulated with plate bound anti-CD3 (1 μg/ml) and soluble CD28 (2 μg/ml) Abs. The cells were cultured under Th2 polarizing conditions (IL-4: 20 ng/ml, IL-2: 100 U/ml, anti-IFN-γ Ab: 5 μg/ml) for 4d at 37 °C/5% CO_2_. The cultures were replenished with polarizing medium for additional 2d. Before harvesting, cells were treated with PMA (40 nM) and ionomycin (1 μM) for 2 h. Later, cultures were treated with brefeldinA (10 μg/ml) for 3 h to block cytokine secretion. CaeA was present in the cultures from the initiation of the experiments. The percentage of Th2 cells was enumerated by intracellular expression of IL-4 and GATA-3 by flowcytometry.

### Flowcytometric analysis of T cells

The cells were washed in FACS buffer (PBS containing 1% FBS) twice after harvesting on final day. For surface staining of CD4 T cells, cells were suspended in FACS buffer containing pacific blue-anti-CD4 Abs (100 μl) and incubated at 4 °C/30 min in dark. The cells were washed twice with FACS buffer and fixed in paraformaldehyde (4%) 200 μl for 4 °C/10 min in dark. The cells were washed with FACS buffer twice. Pelleted cells were resuspended in 200 μl of saponin (0.5%) in PBS for permeabilization. The cells were incubated at RT/15 min in dark. The cells were pelleted and suspended in 50 μl of saponin (0.5% saponin in PBS) containing PE-conjugated anti-GATA-3 and PE/Cy7-labelled anti-IL-4 Abs for 1 h/RT in dark. Later, cells were washed once with saponin. Finally, cells were washed with FACS buffer twice and re-suspended in 300 μl of FACS buffer and acquired in FACSAria and analysed using FACSDiva software.

### Experimental model of asthma

The asthma was induced as mentioned elsewhere^18^ with minor modifications. Briefly, mice were sensitized intraperitoneally (i.p.) with OVA (20 μg) emulsified in alum (4 mg alum) in PBS (100 μl). Booster dose was administered on 12d. Thirteen day onwards, mice were administered CaeA (1, 10 mg/kg bwt) and dexamethasone (Dex) (10 mg/kg bwt) for 12d (13d to 24d) through oral gavage. The animals were aerosol challenged daily with OVA (2%) for 20 min for last 6d (i.e. 18d to 23d). On the final day (24d), the animals were aerosol challenged with OVA for 2 h. After 48 h of last challenge, mice were sacrificed. The severity of asthma was demonstrated by lung histopathology and levels of IL-5 and IL-13 and eosinophils in the BAL and lung cells.

### Enumeration of eosinophils in the BAL

Eosinophils were enumerated as reported elsewhere^19^. Briefly, cells (2 × 10^6^) were incubated with the anti-CD16 Ab to block the Fc receptor in staining buffer (1× PBS supplemented with 2% FBS) for 15 min/4 °C. The cells were washed and incubated with anti-CD11b and siglec-F Abs for 15 min/4 °C. Later, cells were washed and resuspended in fixative buffer (1× PBS + 1% paraformaldehyde). The samples were acquired using FACSAria and analysed by FACSDiva software.

### Isolation of lung lymphocytes

Mice were euthanized with anaesthesia (100 mg/kg, sodium pentobarbital, i.p.). The lungs of the animals were perfused through the right ventricle with chilled PBS till they turned white. Later, they were minced and incubated with digestion mixture (collagenase: 1 mg/ml) at 37 °C/30 min. Digested tissues were disrupted with the help of syringe plunger and passed through 70 μm pore size nylon cell strainers. The residual RBCs were lysed by ACK lysis buffer. The resultant single cell suspension was washed 3× with ice cold PBS. Cell number was counted by trypan blue exclusion method. These cells were *in vitro* challenged with OVA to monitor Th2 response.

### Estimation of IgE

Total IgE in the BALF was estimated by sandwich ELISA. Briefly, 96w plates were coated (50 µl/well) with anti-IgE Abs in bicarbonate buffer (pH 9.6) and incubated for 12 h/4 °C. Later, the plates were blocked with BSA (1%, 100 µl/well). ELISA plates were incubated with SNs (50 µl/well) and standard IgE (40–2000 pg/ml) for 12 h/4 °C. These plates were treated with their corresponding biotin conjugated secondary Abs, followed by streptavidin-HRP. Plates were developed using substrate H_2_O_2_ and chromogenic agent OPD (o-phenylenediamine). The reaction was stopped by mixing equal volume of H_2_SO_4_ (7%). Optical density (OD) of the colour developed was measured at 495 nm. Usual steps of incubations and washings with PBS/Tween-20 (0.05%) were followed at each step. The level of Ab was estimated by plotting standard curve using IgE. Values are expressed as ng/ml. The similar procedure was followed for measuring OVA specific IgE in the BALF using appropriate reagents. Ninety-six well plates were coated with OVA (50 μg/ml) followed by incubation with serum, anti-IgE-biotinylated Abs, streptavidin-HRP and OPD. Regular steps of washing were followed after incubation.

### Estimation of cytokines

The IL-4, IL-5 and IL-13 were determined in the culture SNs and BAL fluid by ELISA, according to the manufacturer’s instruction (BD Bioscience, Franklin Lakes, NJ). Briefly, 96w microtitre plates were coated with respective Abs against IL-4, IL-5 or IL-13 in phosphate buffer (pH-9) for 12 h/4 °C. Later, the plates were blocked with BSA (1%) to eliminate nonspecific binding. The plates were incubated with SNs and appropriate cytokines standard for 12 h/4 °C. The plates were treated with respective biotin conjugated secondary Abs, followed by streptavidin-HRP. The colour was developed with the help of substrate H_2_O_2_ and chromogenic agent OPD. OD of the colour developed was measured at 495 nm. Each step was followed by 5 times washing with 1× PBS-Tween20 (0.05%). The cytokine levels were estimated by plotting standard curve using recombinant cytokines and results expressed as pg/ml.

### Estimation of cytokines by Real-Time PCR

RNA from the lung samples was isolated using TRIzol reagent, according to the manufacturer’s instructions (Invitrogen, Carlsbad, CA). RNA was reverse transcribed to cDNA with the help of synthesis kit (Fisher Scientific, Pittsburgh, PA). cDNA was analysed for the expression of IL-5 and IL-13 by Quantifast SYBR Green PCR kit (Qiagen, Hilden, Germany) employing Realplex master cycler (Eppendorf, Hamburg, Germany).

IL-5

Fwd – 5′-AGCACAGTGGTGAAAGAGACCTT-3′,

Rev 5′-TCCAATGCATAGCTGGTGAT-TT-3′

IL-13

Fwd – 5′-GGAGCTGAGCAACATCACACA-3′,

Rev 5′-GGTCCTGTAGATGGCATTGCA-3′

β-actin

Fwd 5′-AGAGGGAAATCGTGCGTGAC-3′,

Rev 5′-CAATAGTGATGACCTGGCCGT-3′

Ct values of experimental samples were normalized against β-actin, and analysis was done by comparative Ct method. Results are represented as relative expression (fold).

### *In vivo* FMT tomographic imaging of animals suffering from asthma

The imaging of animals was performed as described elsewhere^20^. Briefly, animals were injected ProSense 750Ex i.v. under gas anaesthesia (isoflurane), 24 h prior to imaging. Fur of the animals was removed using hair clipper and depilatory cream to minimize the background interference. The anesthetized animals were carefully placed in the imaging cassette, followed by transfer into the chamber in FMT 2500Lx (PerkinElmer Life Sciences, Waltham, MA) in such a position that the lower abdomen faced CCD camera to give maximum resolution. The animals were excited by appropriate laser and the emitted fluorescence was measured. The amount of fluorescence generated was converted and represented as counts, which were directly proportional to pulmonary inflammation. Image processing and analysis was performed by TrueQuant software.

### Histopathology

The lungs were fixed in buffered formalin (10%). Microtome sections of paraffin-embedded lungs were stained with either hematoxylin and eosin (H&E) or periodic acid-Schiff (PAS). To grade the extent of lung inflammation and goblet cell hyperplasia semiquatitative scoring system was used as previously described^21^,^22^. Briefly, to score the inflammatory cell infiltration, cell counts were performed blind based on five point grading system for the following features: 0: normal, 1: few cells, 2: a ring of inflammatory cells 1 cell layer deep; 3: a ring of inflammatory cells 2–4 cells deep, 4: a ring of inflammatory cells of >4 cells deep. Five fields were counted for each slide and mean score was calculated from five animals. For the quantification of goblet cells in the airway, five point grading system was used, 0: <0.5% PAS positive cells, 1: <25%, 2: 25–50%, 3: 50–75% and 4: >75%. Eight fields were counted for each slide and mean score was calculated from 3 animals.

### Statistical analysis

Statistical analysis was performed with the help of GraphPad Prism software. The difference between groups was compared by using two tailed Unpaired Student’s t-test.

## Results

### CaeA inhibits Th2 differentiation by suppressing the GATA-3 expression

**Figure 1:**
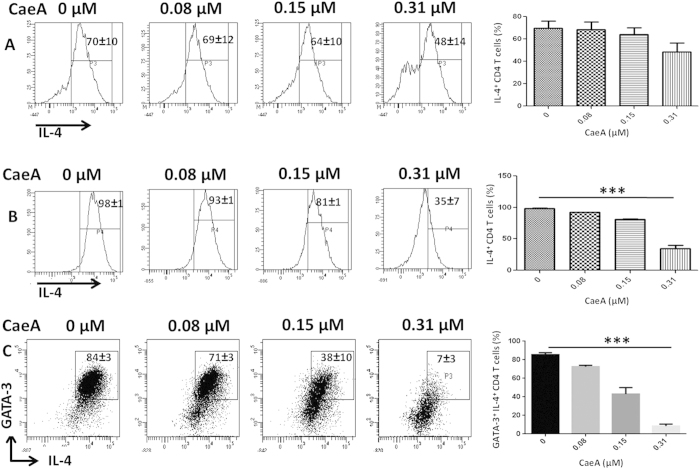
CaeA suppresses Th2 response. (**A**) Th2 cells or (**B,C**) naïve CD4 T cells differentiated under Th2 polarizing conditions were treated with CaeA (0–0.31 μM). The cells were analyzed for the intracellular expression of (**A,B**) IL-4; (**C**) IL-4 and GATA3 on CD4 T cells by flowcytometry. (**A**) The data in the inset of flowcytometer histograms depicts percentage of cells from 3 independent experiments. (**B,C**) The data is representative of 3 independent experiments and mean ± SEM in the inset of flowcytometer histograms and dot plots depicts percentage of cells from duplicate wells. Bar diagram are representative of flowcytometry data. ***p < 0.0005.

IL-4 has been attributed for the differentiation of Th2 cells^23^. Hence, presence of IL-4 is a deciding factor for the generation of Th2 response. The expression of transcription factor GATA-3 is selective for Th2 cells and plays crucial role in the differentiation of naïve CD4 T cells to Th2 cells^24^. Interestingly, we observed inhibition in the intracellular expression of IL-4 in terminally differentiated Th2 cells on treatment with CaeA (Fig. 1A). Similarly, it was noted that naïve CD4 T cells cultured with CaeA under Th2 polarizing conditions also showed significant (p < 0.0005) decrease in the number of IL-4^+^ CD4 T cells (Fig. 1B). Further, these cells also demonstrated substantial (p < 0.0005) downregulation in the expression of GATA-3 in IL-4^+^ CD4 T cells with CaeA treatment (Fig. 1C). These data signify that CaeA suppresses not only polarized Th2 cells but also differentiation of naïve CD4 T cells to Th2 cells by inhibiting the expression of Th2 transcription factor GATA-3.

### CaeA ameliorates the severity of asthma

**Figure 2:**
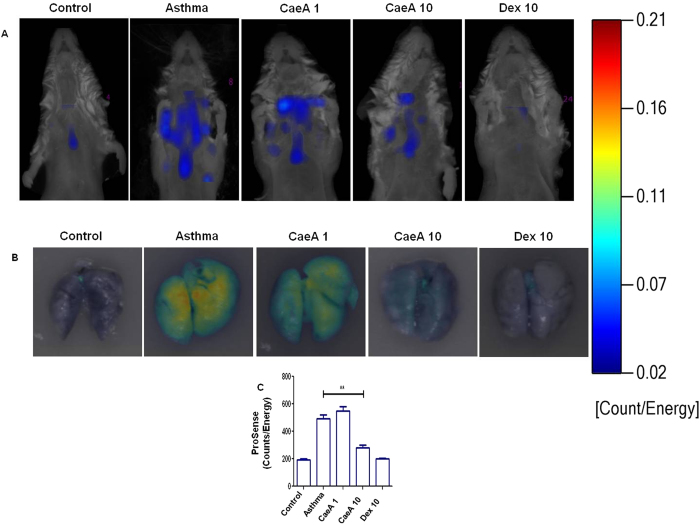
CaeA ameliorates allergic asthma severity. Allergic asthma induced mice were treated with CaeA1 (1 mg/kg bwt) and CaeA10 (10 mg/kg bwt). Animals suffering from asthma receiving Dex10 (10 mg/kg bwt) were kept as a positive control. A placebo (PBS) administered control groups were also kept of either normal mice or suffering from asthma. The disease severity was monitored by measuring lung inflammation. (**A**) Fluorescent tomographic imaging of animals probed with ProSense 750, represents a decrease in disease severity with CaeA and Dex treatment; (**B**) fluorescent reflectance imaging of excised lungs show decreased inflammation by CaeA; (**C**) bar diagram depict decrease in cathepsin activity as measured by ProSense 750 reporter probe. The colour bar on the right side indicates the severity of disease. The data are representative of 3 independent experiments and each bar represents means ± SEM of 6 mice per group. **p < 0.005.

Earlier, we have shown that CaeA can inhibit the secretion of IL-5, a cytokine released by Th2 cells^25^. Th2 cells play a pivotal role in the progression of asthma^26^. Therefore, we hypothesize that treatment with CaeA may attenuate asthma symptoms. Consequently, we next examined *in vivo* effect of CaeA on the OVA induced experimental model of asthma using whole body imager. The fluorescent molecular tomography data very categorically showed substantially lesser disease symptoms in CaeA treated animals than the untreated controls (Fig. 2A). The disease severity and inflammation was measured by cathepsin specific reporter probe ProSense 750. Being a protease-activatable, pan-cathepsin fluorescent *in vivo* imaging agent, it is activated by key disease associated proteases such as cathepsin B, L, S, K, V and D. In this case, cathepsin D, secreted by the eosinophils present in lysosomal granules^20^. Later, *ex vivo* tissue fluorescent reflectance imaging was performed to authenticate the specificity of the signal that originated from the lungs. Excised lungs from the diseased mice showed widespread fluorescence throughout the organ (Fig. 2B). In contrast, CaeA treated animals showed significantly (p < 0.005) lesser fluorescence intensity with the increased dose (Fig. 2B,C). These results were comparable with the positive control group of animals treated with dexamethasone; the drug that is used to treat patients suffering from asthma. These data indicate that CaeA treatment effectively suppresses the asthma symptoms.

### CaeA suppresses airway inflammation and eosinophil counts

**Figure 3:**
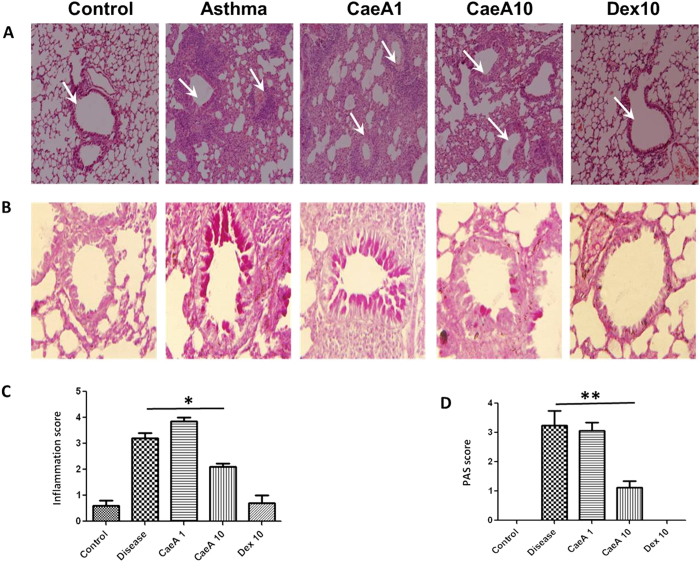
Histology of lung sections. Allergic asthma induced mice were treated with CaeA1 (1 mg/kg bwt) and CaeA10 (10 mg/kg bwt). Animals suffering from asthma receiving Dex10 (10 mg/kg bwt) were kept as a positive control. The experimental and control groups of mice were sacrificed and the lungs were isolated and fixed in formalin solution. Representative microscopic photographs of sections were stained with (**A**) hematoxylin and eosin for the inflammatory cell infiltration analysis and (**B**) periodic acid-Schiff for the quantification of goblet cells. (**A**) Images of lung sections, the arrows denote decrease cellular infiltration by CaeA and Dex. (**B**) Image of lung section shows decrement in number and extent of goblet cell hyperplasia on CaeA and Dex treatment. Bar graph represents semi quantitative scoring of (**C**) inflammatory cell infiltration (mean ± SEM scores were obtained from 5 animals) and (**D**) PAS positive cells (mean±SEM scores were obtained from 3 animals). *p < 0.05, **p < 0.005.

**Figure 4:**
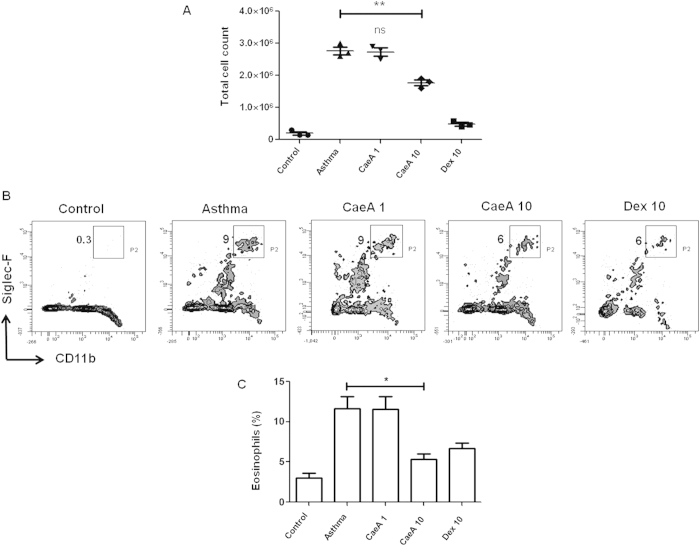
CaeA suppresses the pool of eosinophils in asthma. The asthma induced mice were treated with CaeA and analyzed for cellular infiltration and eosinophil percentage in BAL fluid. (**A**) Dot plot describes the total cell counts, which signify decrease in cellular infiltration by CaeA. Each dot symbolises single mouse and data are represented as mean ± SEM; (**B**) flowcytometry contours depict decrease in the percentage of eosinophils in the BAL fluid of mice treated with CaeA. Value in the inset indicates percentage of eosinophils; (**C**) bar diagram denotes decrease in the percentage of eosinophils by CaeA. Error bar denotes mean ± SEM. Dex was taken as a positive control. The results shown are representative of 3 independent experiments and each bar represents means ± SEM of 6 mice per group. *p < 0.05, **p < 0.005.

To further substantiate our findings illustrated in Fig. 2 and to show its biological significance, we conducted lung histopathology. The microscopic images depicted that the inflammatory response considerably decreased with the increasing dose of CaeA. The lung sections of diseased animals clearly represented augmented infiltration of immune cells. Whereas, CaeA treated animals exhibited less infiltration (Fig. 3A). To support the above data, PAS staining of the sections was conducted to examine the goblet cell hyperplasia in the lung tissue. We distinctively observed decline in the extent of mucus secretion and number of mucus secreting cells on CaeA treatment (Fig. 3B). The observations were further confirmed by semiquantitative scoring of the inflammatory cells infiltration and PAS positive cells showing significant reduction in the airway inflammation (p < 0.05) and goblet cell hyperplasia (p < 0.005) on treating with CaeA, as compared to diseased animals without treatment (Fig. 3C,D). We also analyzed the amount of inflammation by computing the total cell number in BAL fluid. The result shows that CaeA treated animal exhibited significantly (p < 0.005) lesser number of cells in the BAL fluid (Fig. 4A). As observed earlier, CaeA could regress the severity of disease symptoms indicated by lesser fluorescence intensity measured by FMT (Fig. 2A,B). Infiltration of eosinophils is a characteristic feature of asthma^27^. Hence, we examined the anti-asthmatic effect of CaeA by enumerating the eosinophils in the BAL fluid. The data showed that the percentage of eosinophils in CaeA treated animals was significantly lower (p < 0.05) than that of diseased animals and corresponded to animals treated with dexamethasone (Fig. 4B,C). These observations are consistent with *in vivo* imaging results using FMT. Together, these data affirms prominent reduction in the inflammation in lungs on treating with CaeA.

### Animals suffering from asthma on treatment with CaeA exhibited significant inhibition in the Th2 response

**Figure 5:**
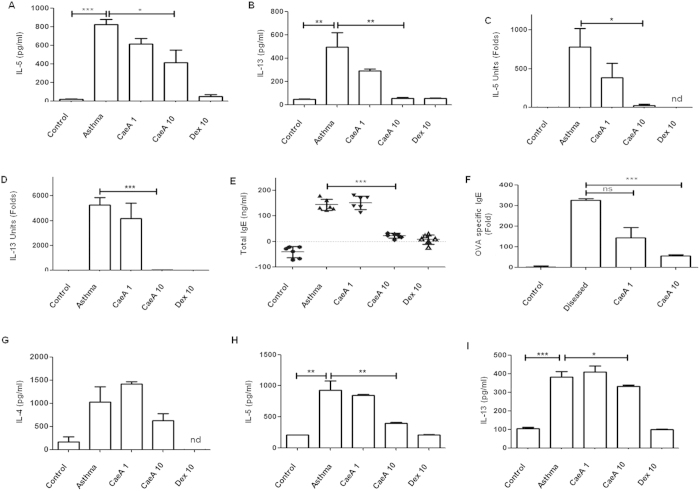
CaeA treated asthma mice exhibit downregulation of Th2 response. Allergic asthma induced mice were treated with CaeA1 (1 mg/kg bwt) and CaeA10 (10 mg/kg bwt). Animals suffering from asthma receiving Dex10 (10 mg/kg bwt) were kept as a positive control. A placebo (PBS) administered control groups were also kept of either normal mice or suffering from asthma. The animals were sacrificed and Th2 response was monitored. (**A,B**) Bar diagram depicts the reduced levels of IL-5 and IL-13 by ELISA in the BALF; (**C,D**) RT-qPCR data support the decreased expression of IL-5 and IL-13 in lungs; (**E**) dot plots represent decline in total IgE in BALF; each dot denotes a single mouse; (**F**) antigen specific IgE content in BALF; (**G**–**I**) ELISA data show diminished production of IL-4, IL-5 and IL-13 by lymphocytes in the SNs on *in vitro* challenged with OVA. Error bar represents mean ± SEM. The results shown are from 2–3 independent experiments and each bar represents means ± SEM of 6 mice per group. nd: not detected. *p < 0.05, **p < 0.005, ***p < 0.0001.

Th2 cells play a fundamental part in the pathogenesis of asthma. Upregulation of Th2 cytokines is the characteristic feature of allergic asthma^28^. Neutralization of Th2 cytokines IL-4, IL-5 and IL-13 leads to decrease in the disease symptoms^29^,^30^,^31^. Hence to substantiate our results that CaeA diminishes disease symptoms (Fig. 4), we demonstrated that CaeA treatment significantly suppressed the Th2 response, in the animals suffering from asthma, as evidence by decreased levels of IL-5 and IL-13 expression in both BAL fluid (Fig. 5A,B) and lung tissues (Fig. 5C,D), as compared to untreated group. The decrease in the cytokines level correlated with the increase in the dose of CaeA. The animals fed with a dose of 10 mg/kg bwt of either CaeA or Dex, expressed nearly same results.

Th2 cells promote the generation of IgE. Elevated level of IgE is an established hallmark for asthma progression. Intriguingly, there was substantial (p < 0.001) decline in the level of total and OVA specific IgE (Fig. 5E,F). Furthermore, we also observed decrease in the yield of IL-4, IL-5 (p < 0.01) and IL-13 (p < 0.05) released by splenic lymphocytes upon *in vitro* exposure to OVA (Fig. 5G–I). These data categorically signify that CaeA ameliorates asthma severity by suppressing Th2 response.

## Discussion

Asthma is an inflammatory disease characterized by airway constriction and hyper reactivity to various allergens. Th2 cells orchestrate the development and progression of allergic asthma^25^,^32^. Th2 cells produce mainly IL-4, IL-5, and IL-13. These cytokines play an important role in the activation of eosinophils and mast cells and elevate the levels of IgE Abs^33^,^34^. IL-4 is responsible for isotype class switching of B lymphocytes to produce IgE^35^. In addition, it stimulates the expression of mucin and eotaxim by fibroblasts^36^,^37^. These factors contribute to the pathogenesis of asthma. Therefore, suppressing Th2 response is a worthy remedy for controlling asthma. Consequently, we started the current study since our preliminary experiments suggested that CaeA can efficiently suppress the Th2 response^19^. Further, many patients suffering from asthma either does not respond to the existing drugs or become refractory to them^9^,^10^. Accordingly, to overcome this problem, it becomes an urgent need to discover newer drugs.

Keeping in view of above mentioned facts, we studied the role of CaeA in animals suffering from asthma. Following major findings showed that CaeA significantly ameliorated the disease symptoms as evidenced by (i) decline in the inflammation, as observed through fluorescent molecular tomography by *in vivo* imaging of the mice; (ii) diminution in the lung pathology by histopathological analysis; (iii) reduced levels of Th2 cells and related cytokines IL-4, IL-5, IL-13; (iv) decrease in the IgE and eosinophils; (v) downregulation in the expression of GATA-3, confirming the authenticity of the CaeA action on Th2 cells.

Airway inflammation has been widely demonstrated in asthma individuals, and an association between the extent of inflammation and the disease severity has been reported^38^. When an asthma patient inhales allergen, it leads to an early allergic inflammatory response^39^,^40^. Interestingly, we noticed that CaeA treatment significantly attenuated the inflammatory response in the mice suffering from asthma. Further, the total number of cells infiltrated into the BAL was also reduced.

Infiltration of eosinophil is a characteristic feature of asthma. IL-5 secreted by Th2 cells play a critical role in the differentiation, maturation and recruitment of eosinophils^27^,^41^. IL-5 is also responsible for the release of chemical mediators (superoxides) from the eosinophils^42^. Therefore, IL-5 has been implicated in the disease pathogenesis and makes it to be a crucial target for treating allergic diseases^43^. On the other hand, IL-13 plays an important role in the smooth muscle hyperplasia and subepithelial fibrosis^44^. Presence of Th2 cytokines in BAL fluid and bronchial submucosa can be observed in asthma individuals^45^,^46^,^47^. Intriguingly, we observed that asthma mice treated with CaeA exhibited significant reduction in IL-5 and IL-13 levels in the BALF, as well as in the lungs. Further, infiltration of eosinophils in the lungs was also reduced, as was clearly depicted in decrease in fluorescence intensity and histopathological analysis of the lungs. We considered that this effect is due to CaeA mediated inhibition of IL-5 and IL-13 in the lungs. The elevated levels of IgE against allergen is a biomarker of asthma^48^. CaeA treatment suppressed the IgE levels in asthma animals. Our studies on *ex vivo* antigen challenged total lung cells also showed decreased expression of IL-4, IL-5 and IL-13. Furthermore, we also established that CaeA not only inhibits the differentiated Th2 cells but also suppresses the naïve T cells from differentiating into Th2 cells. These results very categorically confirm that CaeA eases the asthma severity by suppressing Th2 response. Although, an acute and chronic form of asthma follows similar effector pathway, efficiency of CaeA has yet to be tested in regressing chronic asthma.

The expression of GATA-3 is a hallmark for the Th2 cell lineage^24^. It is fundamental in the exhibition of Th2 cytokines^49^. Hence, it was necessary for us to substantiate our data of suppression of Th2 response by CaeA by monitoring the expression of GATA-3. Interestingly, we noted remarkable downregulation in the expression of GATA-3 in the Th2 cells treated with CaeA. Thus, emphatically establishing the inhibitory role of CaeA on Th2 cells.

In essence, our study indicates that CaeA exhibits anti-asthmatic activity by suppressing inflammatory response in the lungs. This reduction was due to suppression of Th2 response in airway mucosal system. Finally, these results suggest potential role of CaeA in future for the treatment of allergic asthma.

## Additional Information

**How to cite this article**: Kujur, W. *et al.* Caerulomycin A inhibits Th2 cell activity: a possible role in the management of asthma. *Sci. Rep.* **5**, 15396; doi: 10.1038/srep15396 (2015).
